# The prognostic impact of the platelet distribution width-to-platelet count ratio in patients with breast cancer

**DOI:** 10.1371/journal.pone.0189166

**Published:** 2017-12-07

**Authors:** Hideya Takeuchi, Miyuki Abe, Yohei Takumi, Takafumi Hashimoto, Ryoji Kobayashi, Atsushi Osoegawa, Michiyo Miyawaki, Tatsuro Okamoto, Kenji Sugio

**Affiliations:** Department of Thoracic and Breast Surgery, Oita University Faculty of Medicine, Oita, Japan; University of North Carolina at Chapel Hill School of Medicine, UNITED STATES

## Abstract

Activated platelets promote tumor cell growth, angiogenesis, and invasion. Platelet activity can be inferred by platelet volume indices (PVIs), which include platelet distribution width (PDW), mean platelet volume (MPV), platelet distribution width-to-platelet count ratio (PDW/P), and mean platelet volume-to-platelet count ratio. Platelets and platelet-related markers, such as the platelet-to-lymphocyte ratio, have been found to be significant prognostic factors in patients with breast cancer. However, the role of PVIs for predicting survival in breast cancer remains unknown; hence, we performed this retrospective analysis of 275 patients with breast cancer. PVIs were compared with clinicopathological variables, and were assessed to identify independent indicators associated with disease-free survival (DFS) using the Cox proportional hazards model. An elevated PDW/P significantly correlated with age and HER2 status. Univariate analysis revealed that elevated PDW, MPV, and PDW/P as well as tumor size, nuclear grade, and lymph node involvement were significantly associated with inferior DFS rates (tumor size: p<0.01; nuclear grade, lymph node involvement, PDW, MPV, and PDW/P: p<0.05). On multivariate analysis, a large tumor size and elevated PDW/P were significant prognostic factors for DFS, with hazard ratios of 3.24 (95% confidence interval [CI]: 1.24–8.47) and 2.99 (95% CI: 1.18–7.57), respectively (p<0.05). Our study is the first to reveal that an elevated PDW/P significantly reduces DFS in patients with breast carcinoma. Measuring the PDW/P is simple, relatively inexpensive, and almost universally available using routine blood counts; this makes it an attractive biomarker for improved risk assessment.

## Introduction

Breast cancer is the commonest malignant disease among Japanese women, and is a frequent cause of cancer-related death [1, 2]. The conventional tumor node metastasis (TNM) staging system can predict the prognosis of breast cancer; however, clinical outcomes vary remarkably among patients with similar TNM stages [3]. Although molecular diagnostic tests such as Oncotype Dx and Mammaprint may be used to obtain additional prognostic information and help guide clinical therapies in Europe and the United States, the Japanese National Health Insurance does not sustain their use in routine clinical practice owing to the high cost and regional availability of these kits [4]. Thus, identifying prognostic breast cancer biomarkers that are low-cost and easily obtainable via routine blood counts is of great importance.

Platelet activation has been shown to be common in cardiovascular diseases such as acute ischemic stroke, myocardial infarction, and renal artery stenosis [5]. Recently, more attention has been focused on the clinical significance of platelet activation in several malignancies [6]. Platelet-derived growth factor-receptor (PDGF-R) is involved in cancer invasion and metastases. Indeed, elevated PDGF and PDGF-R levels in several tumor tissues are negative prognostic factors [7–11]. We have also shown that platelet-related markers, such as the platelet-to-lymphocyte ratio (PLR), are significant prognostic factors in patients with breast cancer [12,13].

Larger platelets store more granules and receptors, and adhere more rapidly than smaller ones. Thus, the platelet’s activity is more accurately represented by their size, not count [14]. Platelet size can be inferred from the platelet volume indices (PVIs), including the platelet distribution width (PDW), mean platelet volume (MPV), platelet distribution width-to-platelet count ratio (PDW/P), and mean platelet volume-to-platelet count ratio (MPV/P). PDW reflects the variation and heterogeneity in platelet size, and is used in the differential diagnosis of thrombocytopenia [15]. MPV is an indicator of activated platelets and is linked to various inflammatory conditions [16]. Both PDW and MPV are routinely measured by automated common blood count analyzers. Recent studies revealed that MPV and MPV/P levels are associated with poor prognoses in esophageal, breast, hepatocellular, and lung carcinomas [17–21]. However, to the best of our knowledge, the prognostic values of PDW and PDW/P in patients with breast cancer has not been reported.

Hence, the purpose of our study was to evaluate the effect of the PVIs on the DFS rates of patients with localized breast cancer to determine their prognostic significance.

## Patients and methods

### Patients

This was a retrospective study of 327 patients with histologically confirmed breast cancer who underwent surgery at the Department of Thoracic and Breast Surgery, Oita University Faculty of Medicine between April 2006 and December 2016. Fifth-two patients who were excluded from our analysis because of distant metastases at initial presentation (n = 8), carcinoma in situ (n = 25), bilateral breast carcinoma (n = 7), male breast carcinoma (n = 2), medical anticoagulant treatment (n = 1), and insufficient laboratory data (n = 9). Ultimately, 275 patients with localized breast cancer were eligible for this study. As previously described in detail [12, 13], adjuvant therapy was administered according to the St. Gallen recommendations [22]. Follow-up care was performed at regular intervals (3-month intervals during years1-5 and at 6-month intervals during years 5–10 post-diagnosis). Follow-up investigations included clinical examinations, laboratory data analyses (carcinoembryonic antigen and carbohydrate-antigen 15–3 levels), and radiological assessment (computed tomography and mammography) every 12 months during years 1–10 post-diagnosis.

### Pathological characteristics

As described previously [12, 13], pathological data were reviewed to determine the tumor size, nuclear grade, lymph node status, hormone receptor status and human epidermal growth factor receptor 2 (HER2) status. Estrogen and progesterone receptor statuses were evaluated via immunohistochemistry (IHC). Tumors with nuclear expression scores above 0 were considered positive. HER2 status was assessed via IHC or fluorescence in situ hybridization and was considered positive upon obtaining either an IHC score of 3 or at least a 2.2-fold stronger HER2 signal relative to the CEP-17 signal in the tumor cells [23].

### PVI measurement

Blood samples were obtained via peripheral venous puncture before the initiation of any treatment modality. PVIs were measured routinely with an automatic nephelometer (XN-9000; Sysmex Corporation, Kobe, JAPAN) according to the manufacturer’s instructions.

The PDW/P and MPV/P were calculated as the absolute platelet distribution width and platelet mean volume count, respectively, divided by the platelet count (10^4^/mL). The ideal cutoff value for PVI was determined by receiver operating characteristics (ROC) curve analysis.

### Statistical analysis

The primary endpoint of the study was DFS defined as the interval between the date of initial treatment and the first observation of disease relapse. The descriptive statistics are presented as means ± standard deviation. The between-group differences were determined using Student’s *t*-tests.

Survival curves were estimated using the Kaplan–Meier method. All variables were assessed using a Cox proportional hazards model to identify any independent variables associated with DFS. Hazard ratios (HRs) estimated using Cox analysis were reported as relative risks with their corresponding 95% confidence intervals (CIs). All statistical analyses were performed using EZR (Saitama Medical Center, Jichi Medical University, Saitama, Japan), a graphical user interface for R (The R Foundation for Statistical Computing, Vienna, Austria). EZR is a modified version of R Commander designed to include the statistical functions frequently used in biostatistics [24]. A p-value <0.05 was considered significant.

### Data collection and ethics compliance

This retrospective study, including the opt-out consent method, was approved by the institutional ethics review board (the clinical research board of Oita University, institutional ID: 1294). All medical data from the participants were anonymized and compiled. The study plan and choice to freely refuse participation were announced through the hospital bulletin at the Oita University Faculty of Medicine. Patients were considered to have consented to the study if they did not refuse participation.

## Results

### Patients’ characteristics

The basic characteristics of the enrolled patients are summarized in Table 1. The median age was 64.5 (range: 31–92 years) at the time of diagnosis. Patients were divided into two groups with regards to each of PDW, MVP, PDW/P, and MVP/P according to the cutoff values established by ROC analysis.

**Table 1:** Basic characteristics of the enrolled patients.

Variables	No. (%)
**Age (years)**	
<50	33 (12)
≥50	242 (88)
**Tumor size (mm)**	
<20	179 (65)
≥20	96 (35)
**Nuclear grade**	
1	121 (44)
2, 3	141 (51)
Unknown	13
**Lymph node involvement**	
Negative	207 (75)
Positive	68 (25)
**Estrogen receptor**	
Negative	69 (25)
Positive	206 (75)
**Progesterone receptor**	
Negative	114 (42)
Positive	161 (58)
**HER2**	
Negative	230 (84)
Positive	39 (14)
Unknown	6
**PDW**	
<15.3	227 (83)
≥15.3	48 (17)
**MPV**	
<9	58 (21)
≥9	217 (79)
**PDW/P**	
<0.59	169 (62)
≥0.59	106 (38)
**MPV/P**	
<0.35	62 (23)
≥0.35	213 (77)

Abbreviations: No, number; HER2, human epidermal growth factor receptor 2; PDW, platelet distribution width; MPV, mean platelet volume; PDW/P, platelet distribution width-to-platelet count ratio; MPV/P, mean platelet volume-to-platelet count ratio

ROC analysis showed that the optimal cutoff values for DFS were 15.3, 9.0, 0.59, and 0.35 for the PDW, MPV, PDW/P, and MVP/P, respectively (Table 2).

**Table 2:** Receiver operating characteristics analyses of platelet volume indices in breast cancer patients.

Variables	Cut-off value	AUC (95%CI)	Specificity	Sensitivity
**PDW**	15.3	0.61 (0.47–0.74)	0.85	0.4
**MPV**	9.0	0.62 (0.49–0.76)	0.79	0.48
**PDW/P**	0.59	0.58 (0.46–0.7)	0.64	0.6
**MPV/P**	0.35	0.56 (0.41–0.7)	0.79	0.4

Abbreviations: AUC, area under the curve; PDW, platelet distribution width; MPV, mean platelet volume; PDW/PLT, platelet distribution width to platelet count ratio; MPV/PLT, mean platelet volume to platelet count ratio; CI, confidence interval.

The associations between PVIs and clinicopathological variables are shown in Tables 3 and 4. The PDW correlated with tumor size, estrogen receptor status, and progesterone receptor status. The PDW/P correlated with age and HER2 status (p<0.05).

**Table 3:** Association between platelet volume indices and clinicopathological factors in patients with breast cancer.

Variables	PDW			MPV		
	Average	SD	p-value	Average	SD	p-value
**Age (years)**						
<50	12.35	2.67	0.60	9.20	1.46	0.009
≥50	12.11	2.33		9.83	1.28	
**TS**						
<20	11.87	2.23	0.008	9.81	1.21	0.32
≥20	12.66	2.55		9.64	1.50	
**NG**						
1	12.20	2.37	0.81	9.73	1.32	0.77
2, 3	12.11	2.41		9.77	1.33	
**LN**						
(–)	12.04	2.28	0.20	9.79	1.30	0.46
(+)	12.47	2.62		9.65	1.40	
**ER**						
(–)	12.64	2.76	0.043	9.64	1.49	0.40
(+)	11.98	2.20		9.80	1.26	
**PgR**						
(–)	12.50	2.62	0.039	9.59	1.44	0.07
(+)	11.90	2.15		9.87	1.22	
**HER2**						
(–)	12.25	2.41	0.08	9.71	1.34	0.20
(+)	11.54	2.05		10.01	1.16	

**Table 4:** Association between platelet volume indices and clinicopathological factors in patients with breast cancer.

Variables	PDW/P			MPV/P		
	Average	SD	p-value	Average	SD	p-value
**Age (years)**						
<50	0.506	0.14	0.049	0.382	0.12	0.009
≥50	0.616	0.32		0.504	0.27	
**TS**						
<20	0.603	0.32	0.96	0.502	0.27	0.27
≥20	0.602	0.28		0.466	0.23	
**NG**						
1	0.607	0.23	0.85	0.492	0.20	0.89
2, 3	0.599	0.36		0.487	0.31	
**LN**						
(–)	0.609	0.32	0.56	0.503	0.28	0.12
(+)	0.584	0.26		0.448	0.16	
**ER**						
(–)	0.629	0.43	0.40	0.487	0.37	0.91
(+)	0.594	0.24		0.491	0.21	
**PgR**						
(–)	0.628	0.39	0.25	0.489	0.33	0.96
(+)	0.585	0.22		0.490	0.19	
**HER2**						
(–)	0.619	0.32	0.038	0.499	0.27	0.21
(+)	0.511	0.20		0.442	0.15	

Abbreviations: TS, tumor size; NG, nuclear grade; LN, lymph node involvement; ER, estrogen receptor; PgR, progesterone receptor; HER2, human epidermal growth factor receptor 2; SD, standard deviation; p, p-value; PDW, platelet distribution width; MPV, mean platelet volume; PDW/P, platelet distribution width to platelet count ratio; MPV/P, mean platelet volume to platelet count ratio.

### Survival

After a median follow-up duration of 45 months, 25 patients (9.1%) had experienced recurrence. Univariate analysis revealed significant impacts of tumor size, nuclear grade, lymph node involvement, PDW, MPV, and PDW/P on DFS. Other variables were not found to be significantly correlated with DFS. Thus, variables that showed a p-value < 0.05 on univariate analysis were subjected to multivariate analysis. On multivariate analysis, large tumor size and elevated PDW/P were significantly correlated with poor prognosis for DFS, with HRs of 3.24 (95% CI: 1.24–8.47, p<0.05), and 2.99 (95% CI: 1.18–7.57, p<0.05), respectively (Table 5).

**Table 5:** Survival analyses of clinicopathological factors and platelet volume indices.

Variables	Univariate analysis		Multivariate analysis	
	Hazard ratio (95% CI)	p-value	Hazard ratio (95% CI)	p-value
**Age(years) (years)**		0.15		
<50	1			
≥50	0.51 (0.20–1.27)			
**Tumor size**				
<20 mm	1	0.00005	1	0.017
≥20 mm	4.43 (1.91–10.27)		3.24 (1.24–8.47)	
**Nuclear grade**				
1	1	0.044	1	0.27
2, 3	2.45 (1.02–5.87)		1.72 (0.66–4.47)	
**LN**				
Negative	1	0.029	1	0.17
Positive	2.41 (1.09–5.3)		1.78 (0.78–4.1)	
**ER**				
Negative	1	0.29		
Positive	0.64 (0.28–1.45)			
**PgR**				
Negative	1	0.84		
Positive	1.09 (0.49–2.41)			
**HER2**				
Negative	1	0.82		
Positive	0.87 (0.26–2.91)			
**PDW**				
<15.3	1	0.049	1	0.48
≥15.3	2.24 (1.0–5.03)		0.62 (0.17–2.31)	
**MPV**				
<9	1	0.049	1	0.18
≥9	0.45 (0.2–1.0)		0.44 (0.13–1.45)	
**PDW/P**				
<0.587	1	0.042	1	0.021
≥0.587	2.3 (1.03–5.11)		2.99 (1.18–7.57)	
**MPV/P**				
<0.35	1	0.11		
≥0.35	0.52 (0.23–1.17)			

Abbreviations: TS, tumor size; NG, nuclear grade; LN, lymph node involvement; ER, estrogen receptor; PgR, progesterone receptor; HER2, human epidermal growth factor receptor 2; SD, standard deviation; p, p-value; PDW, platelet distribution width; MPV, mean platelet volume; PDW/P, platelet distribution width to platelet count ratio; MPV/P, mean platelet volume to platelet count ratio; CI, confidence interval.

**Fig 1:**
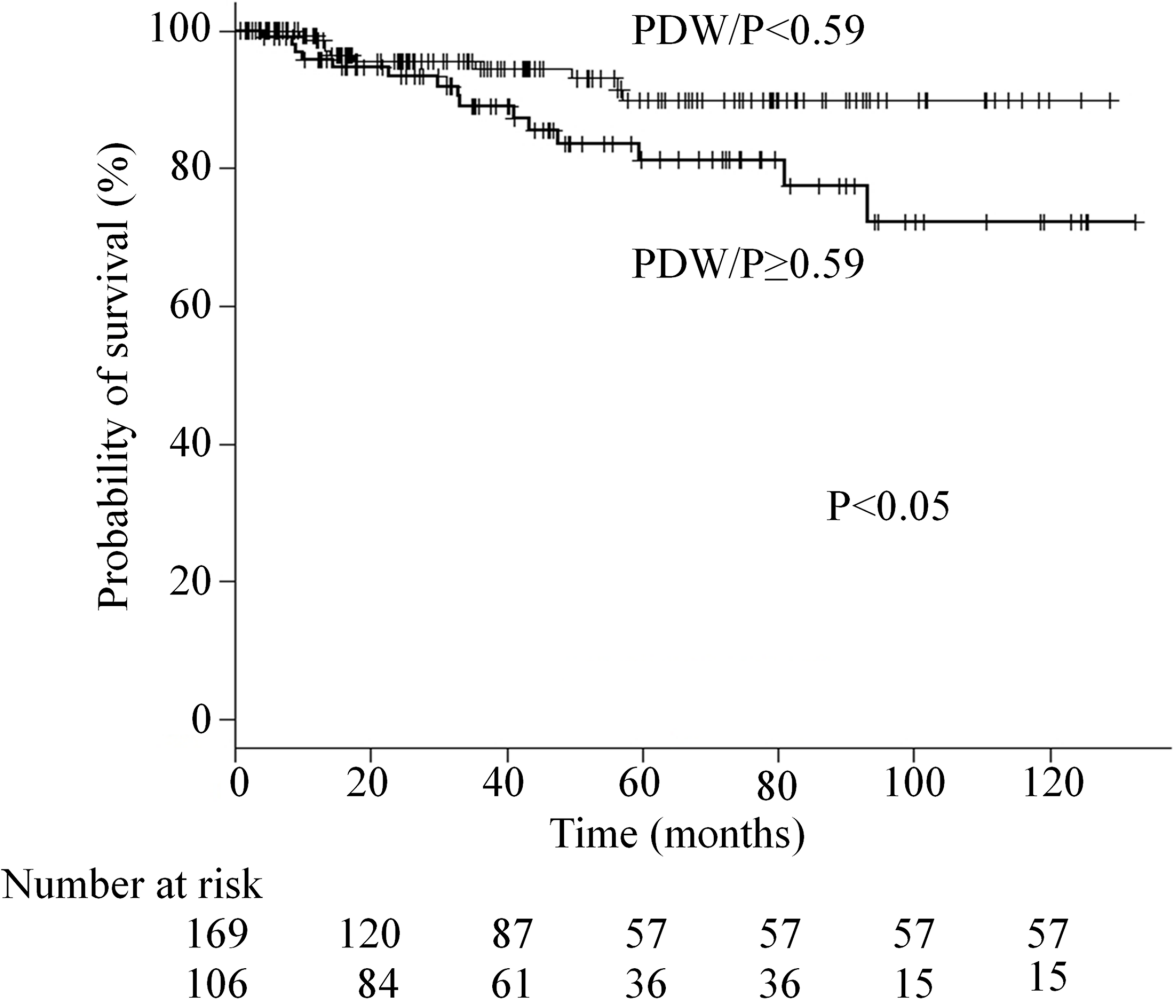
Kaplan-Meier analysis of DFS stratified by the platelet distribution width to platelet count ratio in patients with breast carcinoma.

The DFS rate was significantly lower in the elevated PDW/P group than in the low PDW/P group (5-year survival, 81.3% vs. 89.9%, respectively; p< 0.05) (Fig 1).

## Discussion

Platelets are rich in growth factors such as PDGF [7–11], transforming growth factor -β [25], and platelet -derived endothelial cell growth factor [26]. These platelet-derived growth factors are often produced in large quantities by cancer cells and contribute to their progression [25]. In breast cancer, PDGF beta receptor expression correlates with unfavorable clinicopathological cahracteristics and survival [10]. Kang DW et al. showed that PDGF contributes to the aggressiveness of breast cancer cells by the NFkB signaling pathway [11]. Elevated platelet counts and platelet-related markers, such as the PLR, are associated with a poor prognosis [12,13]. To the best of our knowledge, ours is the first study to demonstrate the value of PDW/P as an independent prognostic factor in breast cancer patients. Our findings may provide important supporting information to inform treatment decisions and predict treatment outcomes.

MPV and PDW are morphometric indices as well as quantitative measures of size distribution and variability of platelets; in general, the two are inversely proportional [27], and can be utilized to differentiate between hypoproductive and hyperdestructive thrombocytopenia, respectively [28]. PDW is a more specific marker of platelet activation, because it does not increase owing to platelet swelling [29]. There are pronounced contraindicatory results regarding the relationship between MPV and prognosis in various cancers. Some studies found that elevated MPV levels significantly correlate with unfavorable prognoses in esophageal [17], breast [18], and hepatocellular [19] carcinoma. On the other hand, reduced MPV and MPV/P were independent predictors of negative prognosis in lung cancer patients [20, 21]. Hence, the published literature regarding the value of MVP as a prognostic factor is inconclusive. PDW may therefore be a more reliable and accurate prognostic marker than MPV in cancer patients.

The reason for the poor prognosis of patients with high PDW/P is unclear. Platelet volume is determined both during megakaryopoiesis and thrombopoiesis. PDW is an indicator of variation and heterogeneity in platelet volume; high values of this index indicates the presence of mature and immature cells simultaneously in circulation. This implies that an increase in PDW may be accompanied by abnormal thrombosis [29] and/or be the result of heterogeneous demarcation of megakaryocytes [30]. Thus, the underlying mechanism might be straightforward. Various pro-inflammatory cytokines such as tumor necrosis factor-*α*, interleukin-1, and interleukin-6 are upregulated concomitantly with tumor progression [31]. These cytokines promote heterogenic megakaryocytic maturation, leading to the production and release of immature platelets with various characteristics and sizes into the circulatory system to meet the increased demands [32]. However, additional investigations are required to better understand the reason why a high PDW/P leads to poor prognosis in breast cancer patients.

The limitations of this study include the relatively small sample size, short follow-up period and single-center design. The retrospective nature of our study may also have caused bias in terms of data selection and analysis. Despite these limitations, our study is the first to reveal that elevated PDW/P is indicative of an unfavorable prognosis in patients with localized breast cancer.

## Conclusions

Despite certain limitations in our study, our data clearly indicate that an increased preoperative PDW/P measurement may represent an independent prognostic factor in patients with localized breast cancer. Measuring the PDW/P is simple, relatively inexpensive, and almost universally available using routine blood counts, making it a readily available biomarker for improved risk assessment. However, our data are preliminary and should be interpreted with caution pending validation by additional clinical studies.

## Supporting information

S1 Fig (XLSX)
